# Human blood-labyrinth barrier model to study the effects of cytokines and inflammation

**DOI:** 10.3389/fnmol.2023.1243370

**Published:** 2023-09-21

**Authors:** Marijana Sekulic, Raoul Puche, Daniel Bodmer, Vesna Petkovic

**Affiliations:** ^1^Department of Biomedicine, University Hospital Basel, University of Basel, Basel, Switzerland; ^2^University Hospital Basel, Clinic for Otorhinolaryngology, Basel, Switzerland

**Keywords:** blood-labyrinth barrier, cytokines, disease model, hearing loss, inflammation

## Abstract

Hearing loss is one of the 10 leading causes of disability worldwide. No drug therapies are currently available to protect or restore hearing. Inner ear auditory hair cells and the blood-labyrinth barrier (BLB) are critical for normal hearing, and the BLB between the systemic circulation and stria vascularis is crucial for maintaining cochlear and vestibular homeostasis. BLB defects are associated with inner ear diseases that lead to hearing loss, including vascular malformations, inflammation, and Meniere’s disease (MD). Antibodies against proteins in the inner ear and cytokines in the cochlea, including IL-1α, TNF-α, and NF-kβ, are detected in the blood of more than half of MD patients. There is also emerging evidence of inner ear inflammation in some diseases, including MD, progressive sensorineural hearing loss, otosclerosis, and sudden deafness. Here, we examined the effects of TNF-α, IL6, and LPS on human stria vascularis-derived primary endothelial cells cultured together with pericytes in a Transwell system. By measuring trans-endothelial electrical resistance, we found that TNF-α causes the most significant disruption of the endothelial barrier. IL6 had a moderate influence on the barrier, whereas LPS had a minimal impact on barrier integrity. The prominent effect of TNF-α on the barrier was confirmed in the expression of the major junctional genes responsible for forming the tight endothelial monolayer, the decreased expression of *ZO1* and *OCL*. We further tested permeability using 2 μg of daptomycin (1,619 Da), which does not pass the BLB under normal conditions, by measuring its passage through the barrier by HPLC. Treatment with TNF-α resulted in higher permeability in treated samples compared to controls. LPS-treated cells behaved similarly to the untreated cells and did not show differences in permeability compared to control. The endothelial damage caused by TNF-α was confirmed by decreased expression of an essential endothelial proteoglycan, syndecan1. These results allowed us to create an inflammatory environment model that increased BLB permeability in culture and mimicked an inflammatory state within the stria vascularis.

## Introduction

**Figure 1:**
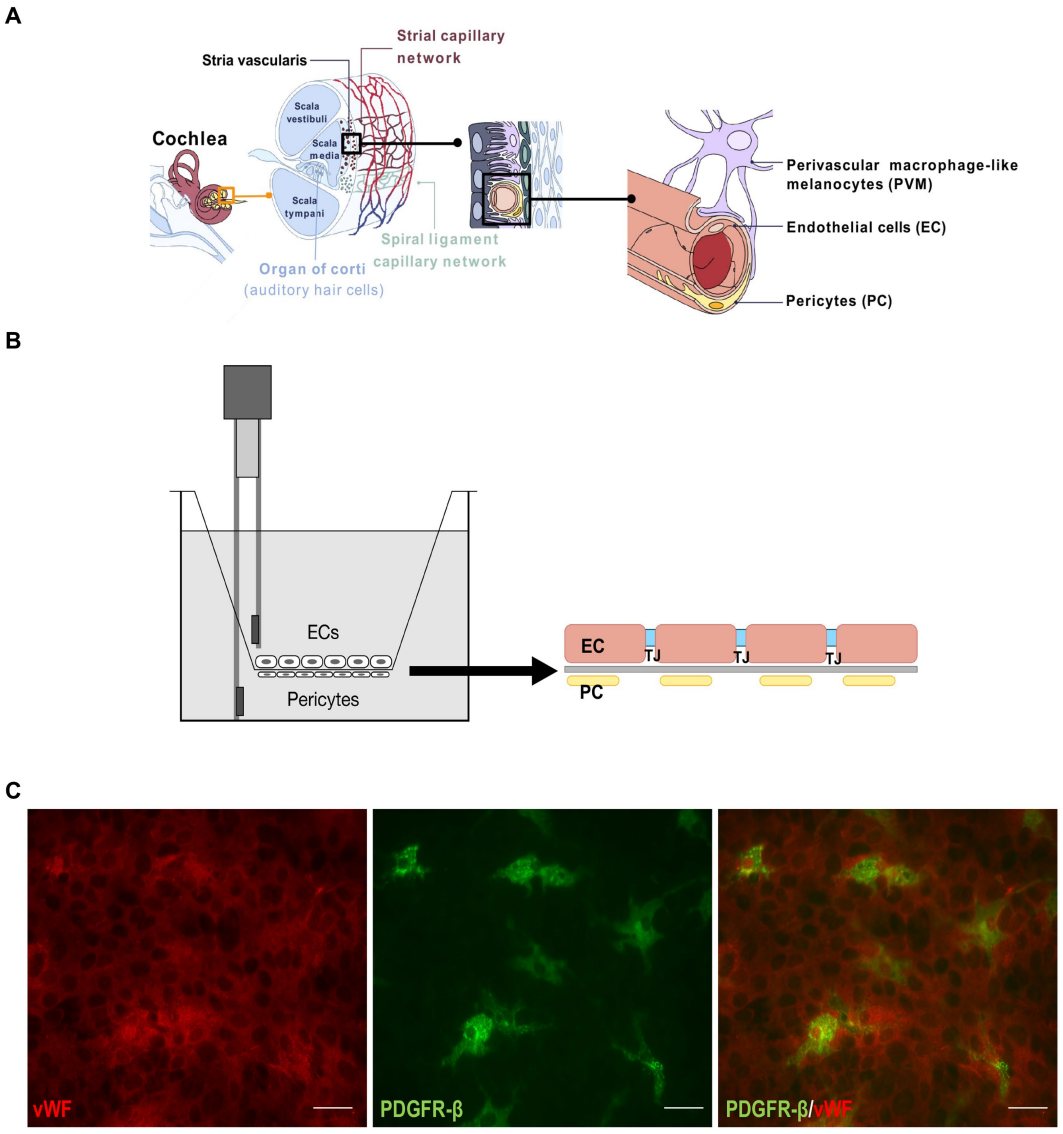
BLB Transwell model. **(A)** Drawing depicting the inner ear components with the magnified representation of stria vascularis capillary network and the blood-labyrinth barrier (last on the right). **(B)** Schematic of the Transwell model. Endothelial cells were cultured on the luminal side of the Transwell membrane and pericytes on the abluminal side, with electrodes to measure TEER. One electrode is submerged in the chamber with endothelial cells and the other in the outer part of the Transwell insert. **(C)** Immunostaining of the endothelial cells on one side of the membrane with von Willebrand factor (red, left) and pericytes stained with PDGFR-β (green, center). A merged image is on the right. Scale bar: 100 μm.

The blood-labyrinth barrier (BLB) in the inner ear refers to the barrier between the vasculature and the inner ear fluids (Juhn et al., 1981). Further on, the blood-inner ear barriers consist of the blood-endolymph barrier (BEB) and the blood-perilymph barrier (BPB) (Zou et al., 2016). The BLB is responsible for maintenance of the inner ear fluid ionic homeostasis and acts as a selective barrier for the entry of various substances into the inner ear. The BLB in the stria vascularis is made of vascular endothelial cells (ECs) surrounded by basement membrane, pericytes (PCs), and perivascular-resident macrophage-like melanocytes (Sakagami et al., 1982; Shi, 2009; Zhang et al., 2012, 2013; Neng et al., 2013a,b) (Figure 1A). ECs build the inner lumen of the blood vessels and are connected by tight junctions, forming a strong barrier between the circulating blood and the rest of the vessel wall. On top of ECs from the endothelial basement side are PCs, the only cells in close contact with ECs. Understanding the dynamics of the BLB is crucial for developing therapeutics for the inner ear to block or enhance the BLB inflammatory response. Though the BLB is well studied in animal models, little data is available in human tissue (Juhn et al., 1982, 2001; Salt et al., 1987).

Recent studies have implicated a loss of BLB integrity in several inner ear pathologies, including acoustic trauma, autoimmune inner ear disease, and presbycusis, as well as Meniere’s disease (MD), Alport syndrome, and Pendred syndrome (Wangemann et al., 2004; Gratton et al., 2005; Shi, 2011; Neng et al., 2013a; Zhang et al., 2013; Goodall and Siddiq, 2015; Heeringa and Köppl, 2019). The inner ear is often the first organ affected in systemic autoimmune diseases, probably due to a mild vascular pathology affecting the ear faster than any other organ system (Goodall and Siddiq, 2015). In autoimmune mice, the primary defects in the inner ear include breakdown of the stria vascularis blood vessels, loss of BLB integrity, loss of endocochlear potential, and hearing loss (Lin and Trune, 1997; Trune et al., 2000). In a guinea pig study recreating aseptic inflammation was found that endolymphatic hydrops may develop due to increased blood-endolymph barrier permeability (Zou et al., 2007). MD is an inner ear disorder influenced by genetic, epigenetic, and environmental factors. The exact etiology and pathophysiology remain unresolved. It is characterized by recurrent vertigo and cochlear impairments marked with sensorineural hearing loss (SNHL), tinnitus, or aural fullness (Espinosa-Sanchez and Lopez-Escamez, 2016; Amanat et al., 2020). Endolymphatic hydrops, characterized by excess endolymph accumulation, was demonstrated in postmortem human temporal bone studies on patients with MD (Hallpike and Cairns, 1938; Merchant et al., 2005). In MD, the BLB is impaired in association with hydrops grade, which has been suggested to be caused by dysfunctional inner ear blood flow exacerbated by a pathological increase in the vascular permeability of the BLB (Foster and Breeze, 2013). Decreased blood flow and increased BLB permeability have also been described in MD patients (Miller et al., 1995; Pakdaman et al., 2016). The BLB dysfunction in the microvasculature of MD patients likely contributes to edematous changes (Ishiyama et al., 2017). MD is a uniquely human disease and there are no animal models that replicate it. Current *in vitro* and animal models used in drug discovery for hearing loss have a very low success rate. There is a gap in establishing human BLB models that would help translate drug discovery data to a human-relevant system and increase the success rate of drug delivery and efficacy.

Antibodies against proteins in the inner ear and cytokines in the cochlea, including interleukin (IL)-1β, tumor necrosis factor alpha (TNF-α), and NF-kβ, were detected in blood from more than 50% of MD patients (Frejo and Lopez-Escamez, 2022). Single cell cytokine profile revealed two clusters of MD patients with differences in IgE levels, immune cell population abundance, including a reduction of CD56dim NK-cells, and changes in cytokine expression (Flook et al., 2023). TNF-α is a cytokine that has multiple effects on various cell types. It regulates inflammatory responses and is known to be involved in the pathogenesis of some inflammatory and autoimmune diseases (Bradley, 2008). TNFα and IL-6 that are produced at the early stages of cochlear inflammatory response. Pro-inflammatory cytokine, IL-6, is upregulated in the early phases of a cochlear inflammatory response and is found to be increased in patients with MD and ototoxic insults (Satoh et al., 2003; Fujioka et al., 2006; Frejo et al., 2018).

In this study we used a Transwell model in which we co-cultured human stria vascularis-derived primary ECs and PCs on each side of the porous membrane. We exposed the ECs to TNF-α, IL-6, and lipopolysaccharide (LPS) and observed their influence on endothelial permeability. These results allowed us to create an inflammatory environment that affected BLB permeability and modeled an inflammatory state within the stria vascularis.

## Methods

### Human tissue isolation

We have established a protocol for the isolation, maintenance, and differentiation of human stria vascularis from human post-mortem tissues obtained from the Pathology Institute in Basel, Switzerland (Ethical permit: EKNZ 2020-01379). Autopsy-derived post-mortem human temporal bones were used as a tissue source due to the paucity of surgical procedures in which healthy stria vascularis tissue could be obtained. Donors were from both genders and ranged in age from 50 to 75 years, five male and one female. Exclusion criteria were prior hearing disorders or loss (with the exception of age-related hearing loss), recent chemotherapy, and any diseases that could affect hearing. The samples were collected an average 11 h post-mortem, but up to 18 h post-mortem. During autopsy, the skulls were opened as usual by removing the skull cap. After removal of the brain, the dura mater at the base of the skull was pulled off. The petrous part of the temporal bone was removed from the base of the skull en bloc using an oscillating saw, so that the middle and inner ear were not opened during removal. The excised bone fragment was immersed in 70% (v/v) ethanol for 30 s and subsequently placed in sterile PBS containing penicillin/streptomycin (100 U/mL) and stored at 4°C until further processing. The temporal bone specimens were stabilized in a temporal bone holder (Storz & Co., Tuttlingen, Germany). An open tympanoplasty approach was performed. After a subtotal mastoidectomy, the superior and posterior canal wall, tympanic membrane, malleus, and incus were removed. The protympanon was enlarged to the petrous segment of the internal carotid artery. Parts of the tympanic segment of the facial nerve and tendon of the m. tensor tympani were dissected to access the full circumference of the cochlea. The promontory bone was blue-lined with a diamond drill until the cochlear turns were visible. The lateral bone of the turns was completely drilled away and the membranous labyrinth of the cochlea carefully extracted.

Tissue was trypsinized with Trypsin–EDTA 0.25% (Sigma cat#T4049) for 5 min, followed by blocking with 10% human serum solution (Sigma cat#H3667) and centrifugation at 1500 rpm for 5 min. Tissue pieces were distributed in wells coated with 0.03 mg/mL fibronectin in DPBS (ScienCell cat#8248) or 0.01 mg/mL poly-L-lysine in sterile water (ScienCell cat#0403) to seed ECs and PCs, respectively, and then rinsed with sterile water and DPBS.

### Cell culture and treatment

The cells were seeded and incubated at 37°C in 5% CO_2_. The growth medium for ECs consisted of 500 mL EC basal medium, 25 mL human serum (Sigma cat#H3667), 5 mL EC growth supplement (ScienCell cat#1052), and 5 mL penicillin/streptomycin solution (ScienCell cat# 0503). The PC growth medium consisted of 500 mL PC basal medium, 10 mL human serum (Sigma cat#H3667), 5 mL PC growth supplement (ScienCell cat#1252), and 5 mL penicillin/streptomycin solution (ScienCell cat#0503). Before the use in experiments cells are cultured and expanded in T25 or T75 flasks coated with appropriate attachment factor. The cells used for the experiments are at the passage 2 or 3, and after expansion in the flask for the seeding on the transwell they are trypsinized, and in the end re-suspended in the appropriate cell medium.

The medium was changed every 2 days. Before placing the cells on the transwell, the cell type was validated at the gene and protein level using immunostaining and gene expression of marker proteins as described below. Treatments were performed with 50 ng/mL TNF⍺, 20 ng/mL IL-6, and 100 ng/mL LPS for 72 h, and controls were treated with vehicle only.

### Real-time PCR

RNA was isolated from collected cells and extracted using the Direct-Zol RNA MiniPrep kit (Zymo Research, Germany, cat#R2050) according to the manufacturer’s instructions. Total RNA (1,000 ng) was reverse-transcribed using a High-Capacity cDNA Reverse Transcription Kit (Applied Biosystems, United States). We analyzed triplicate samples by quantitative PCR on an ABI Prism 7900HT Sequence Detection System (Applied Biosystems, United States) using the Power Sybr Green Master Mix (Applied Biosystems, United States). Primers targeting *GAPDH* were synthesized by Microsynth (St. Gallen, Switzerland) and added at a final concentration of 250 nM per reaction. The full primer sequences used in this study were (5`-3`): ***TJP1*** (*ZO1*): Fw – CAA CAT ACA GTG ACG CTT CAC A, Rev. – CAC TAT TGA CGT TTC CCC ACT C; ***F11R*** (*JAM1*): Fw – ATG GGG ACA AAG GCG CAA G, Rev. – CAA TGC CAG GGA GCA CAA CA; ***OCLN*** (*OCL*): Fw – ACA AGC GGT TTT ATC CAG AGT C, Rev. – GTC ATC CAC AGG CGA AGT TAA T; ***CDH5*** (*CADH5*): Fw – TTG GAA CCA GAT GCA CAT TGA T, Rev. – TCT TGC GAC TCA CGC TTG AC; ***CLDN5***: Fw – CTC TGC TGG TTC GCC AAC AT, Rev. – CAG CTC GTA CTT CTG CGA CA; ***GAPDH***: Fw – GGA GCG AGA TCC CTC CAA AAT, Rev. – GGC TGT TGT CAT ACT TCT CAT GG. The relative quantities of specifically amplified cDNAs were calculated by the comparative threshold cycle method (2–∆∆Ct), and *GAPDH* expression was used as the endogenous reference.

### Transepithelial electrical resistance

Transepithelial electrical resistance (TEER) was measured with a Voltohmmeter EVOM3. After counting the cells using Trypan Blue in the automated cell counter (Bio-Rad, TC20) the cell suspension is placed on each side of the coated transwell membrane in two steps. First, the inserts are flipped upside down and 150 μL pericyte cell suspension is placed on the abluminal side. They are then placed back in the incubator for 3 h to allow cells to settle and attach. After 3 h the inserts are flipped back in their original position and placed in the PC medium containing 24 well slot, then EC cell suspension is added in the luminal side of the insert and cells are allowed 3 h to attach in the small volume of 150 μL and then the rest of the EC medium is added.

For TEER measurements, ECs were seeded at a density of 2 × 10^5^/cm^2^ and grown on membrane inserts (Corning, cat#3470) coated with fibronectin. The PCs were combined with ECs (harvested after 2 and 3 passages and seeded at a density of 1 × 10^5^/cm^2^) and grown on the poly-l-lysin-coated inserts. The untreated Transwells containing vehicle were used as a control and prepared the same way as the ones treated with TNF-α, IL-6, and LPS. Measurements were obtained following the manufacturer’s protocol. Briefly, electrodes were maintained by soaking the tips once a week in a 1% Tergazyme solution for 15 min and rinsing with sterile water, and the same was done just before disinfection and before beginning an experiment. The STX4 electrode were disinfected in 70% ethanol for no more than 5 min, followed by rinsing with medium or PBS. This was followed by measuring the resistance in treated samples and controls. Cells were allowed to attach and rest for 24 h before the first measurement was done and then measurements were performed once per day and three measurements per well. First, electrodes were placed in the culture medium for a few minutes. To measure the blank resistance, the electrode was placed in a transwell insert without cells filled with cell medium. Further the measurements in experimental wells with cells were performed.

After finishing the measurement, electrodes were disinfected with ethanol, rinsed with sterile water, and allowed to air dry. To calculate TEER, the surface area of the Transwell (in cm^2^) was multiplied by the net resistance (the resistance of a blank Transwell covered by cell culture media subtracted from the measured resistance).

### Permeability assay

To test the permeability, 2 μg daptomycin (Sigma, cat#SBR00014) was added to the EC medium for the top chamber, whereas the basolateral bottom chamber contained only PC medium. The untreated Transwells containing vehicle were used as a control and prepared the same way as the TNF-α and LPS-treated Transwells. A 20 μL aliquot was taken from the apical and basolateral chamber reservoirs at defined time points for both the BLB Transwell with cells and control sample: 0, 30, 60, 90, 180, and 440 min. The samples were then evaluated by HPLC to determine daptomycin concentrations at those time points.

### Immunostaining and imaging

For syndecan1 staining endothelial cells were grown on 4-well fibronectin coated glass-bottom dishes (Ibidi, Germany, cat#80426). For EC and PC marker staining cells were co-cultured on each side of the Transwell insert (Corning, cat#CLS3470), EC on the luminal fibronectin coated side and PC on the abluminal poly-l-lysin-coated side. Cells were fixed in 4% paraformaldehyde (Sigma, United States, cat#158127) in PBS (Sigma, United States, cat#P4417), permeabilized with 0.1% Triton X-100 (Sigma, United States, cat#X100) in PBS, and incubated for 1 h at room temperature with anti-von Willebrand factor (vWf, Sigma, United States, cat#F3520), anti-PDGFR-β (Santa Cruz Biotechnology, United States, cat#sc374573), anti-CD138-syndecan1 (Invitrogen, cat#MA53-2600) and ZO-1 (Invitrogen #33-9,100) primary antibodies. Next, samples were washed and incubated with appropriate secondary antibodies (Thermo Fisher, United States, cat#A2124, cat#A-11001, cat#A-11008) in PBS-T for 1 h at room temperature. Samples were washed with PBS and incubated with DAPI for 5 min. The cells were washed with PBS and mounted on microscope slides using Dako Fluorescent Mounting Medium (Dako, Denmark, cat#S3023). Images were captured by a Nikon Eclipse Ti2 inverted widefield microscope. Images were processed and analyzed using Fiji-win 32 software.

### Western blotting

ECs were homogenized in cell lysis buffer (Sigma-Aldrich, St. Louis, MO, United States, cat#C3228) with a protease inhibitor cocktail (Sigma-Aldrich, St. Louis, MO, United States, cat#C3228, cat#P8340) for 1 min on ice. Protein concentrations were determined by the Bradford method using Bio-Rad Protein Assay Dye Reagent Concentrate (Bio-Rad, Switzerland, cat#5000006). Protein concentrations were confirmed using a NanoDrop (Thermo Fisher Scientific, Waltham, MA, United States). Samples were mixed with Laemmli sample buffer (Sigma-Aldrich, St. Louis, MO, United States, cat#S3401), heated at 95°C for 5 min, and then resolved on 4–20% Mini-PROTEAN^®^ TGX^™^ precast protein gels (Bio-Rad, Switzerland, cat#4561096). After electrophoresis, separated proteins were transferred onto polyvinylidene fluoride membranes. Membranes were incubated with 5% non-fat dry milk dissolved in PBS-T for 1 h at room temperature to block non-specific protein binding site. Next, the membranes were washed with PBS-T (3 × 10 min) before being incubated overnight at 4°C in 5% non-fat dry milk in PBS with the primary antibody anti-CD138 (syndecan1) (Invitrogen, cat#MA53-2600). The membranes were then washed with PBS-T (3 × 10 min) and incubated for 1 h at room temperature with the appropriate horseradish peroxidase-conjugated secondary antibody. After washing, immunoreactive protein bands were visualized using Super Signal West Dura Extended Duration Substrate (Thermo Fisher Scientific, Waltham, MA, United States, cat#34076). An anti-B-ACTIN antibody was used as a control to demonstrate equivalent protein loading. The intensity of the immuno-positive bands was determined using Fiji-win 32 software, capturing the identical regions on each blot and deducting the background signal. The intensity of all proteins of interest was normalized to the intensity of B-ACTIN in the same sample.

### Statistical analysis

Statistical analyses were performed in GraphPad Prism software (San Diego, CA, United States). Multiple groups were compared by two-way analysis of variance, and two groups were compared by an unpaired Student’s *t*-test. Data were confirmed to be normally distributed by a Shapiro–Wilk test. *p*-values <0.05 were considered significant.

## Results

### TNF-α, IL6, and LPS disrupt the endothelial layer integrity

**Figure 2:**
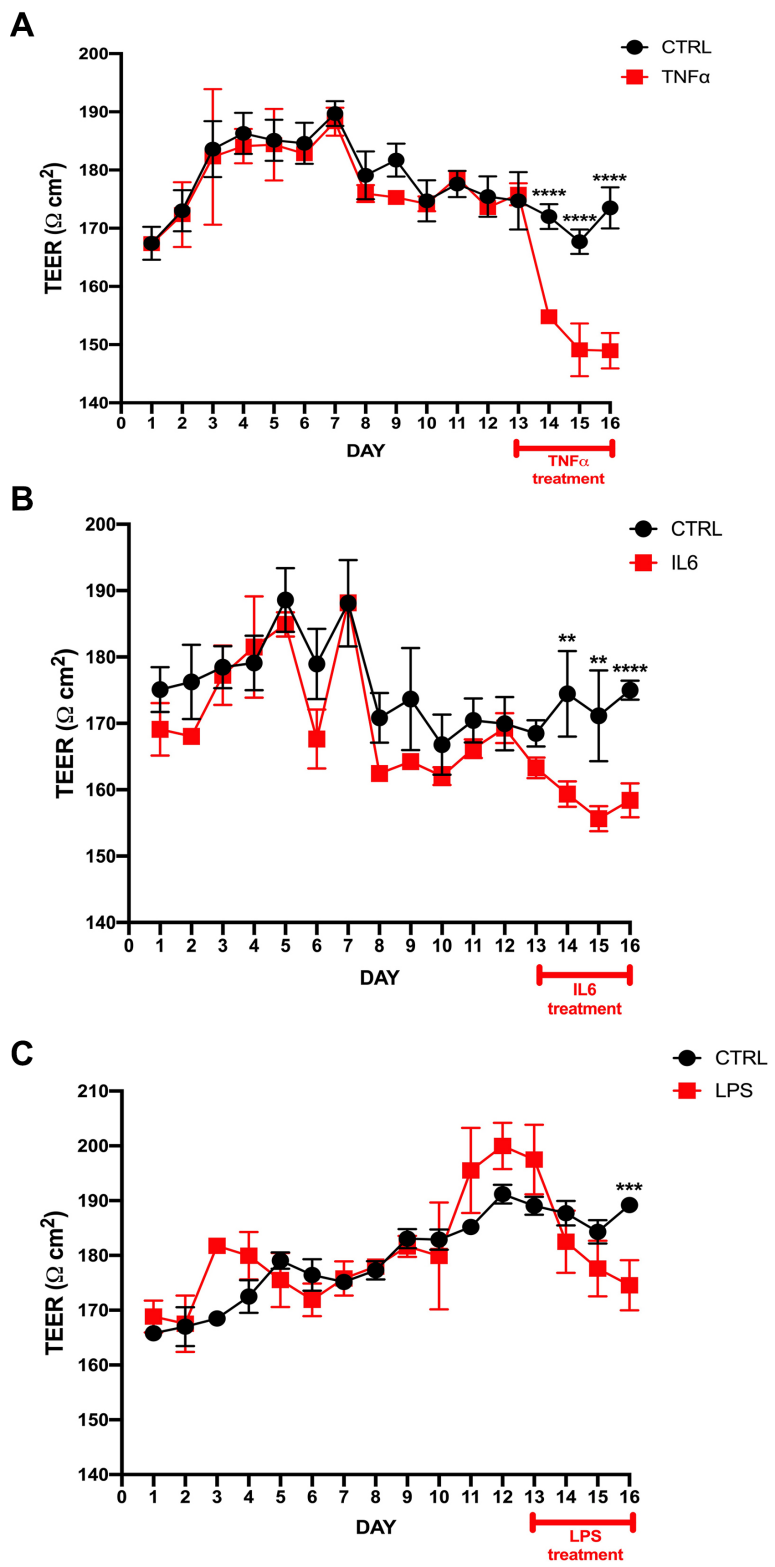
TEER measurement in the BLB Transwell treated with TNF-α, IL-6, and LPS. **(A)** TEER values after seeding endothelial cells and pericytes on a Transwell insert with a polyester membrane. The 50 ng/mL TNF-α treatment started on day 13 and continued over the next 72 h. TEER measurements were performed once per day, with first measurement starting 24 h after seeding and labeled as day 1. TEER measurement was done by 3 consecutive repetitions for each individual transwell. The control cells were treated only with vehicle. The difference between the treatment and control was observed within the first 24 h of treatment. ****p < 0.0001, *n* = 6. **(B)** TEER values after IL-6 treatment vs. control show a significant drop post-treatment. ****p < 0.0001, **p < 0.01, *n* = 6. **(C)** LPS treatment results in significant differences only on the last treatment day compared to control. ***p < 0.001, *n* = 6. Data are presented as mean ± SD.

While culturing the ECs and PCs on each side of the Transwell membrane (Figures 1B,C), daily TEER measurements were performed until the values reached a stable threshold. Upon reaching stable values, TNF-α, IL-6, and LPS were added. TNF-α had the greatest impact on barrier integrity, which was reflected in a significant drop in TEER values in treated Transwells from the first 12 h after the treatment begin, which consistently continued throughout the treatment period (Figure 2A). IL-6 treatment had similar trend as TNF-α, but with less pronounced, though still significant, differences between the treatment and control (Figure 2B). LPS treatment had a modest impact on barrier integrity, showing significant differences between the control and treatment only on the last treatment day (Figure 2C).

### Junctional gene expression is affected by TNF-α and LPS

**Figure 3:**
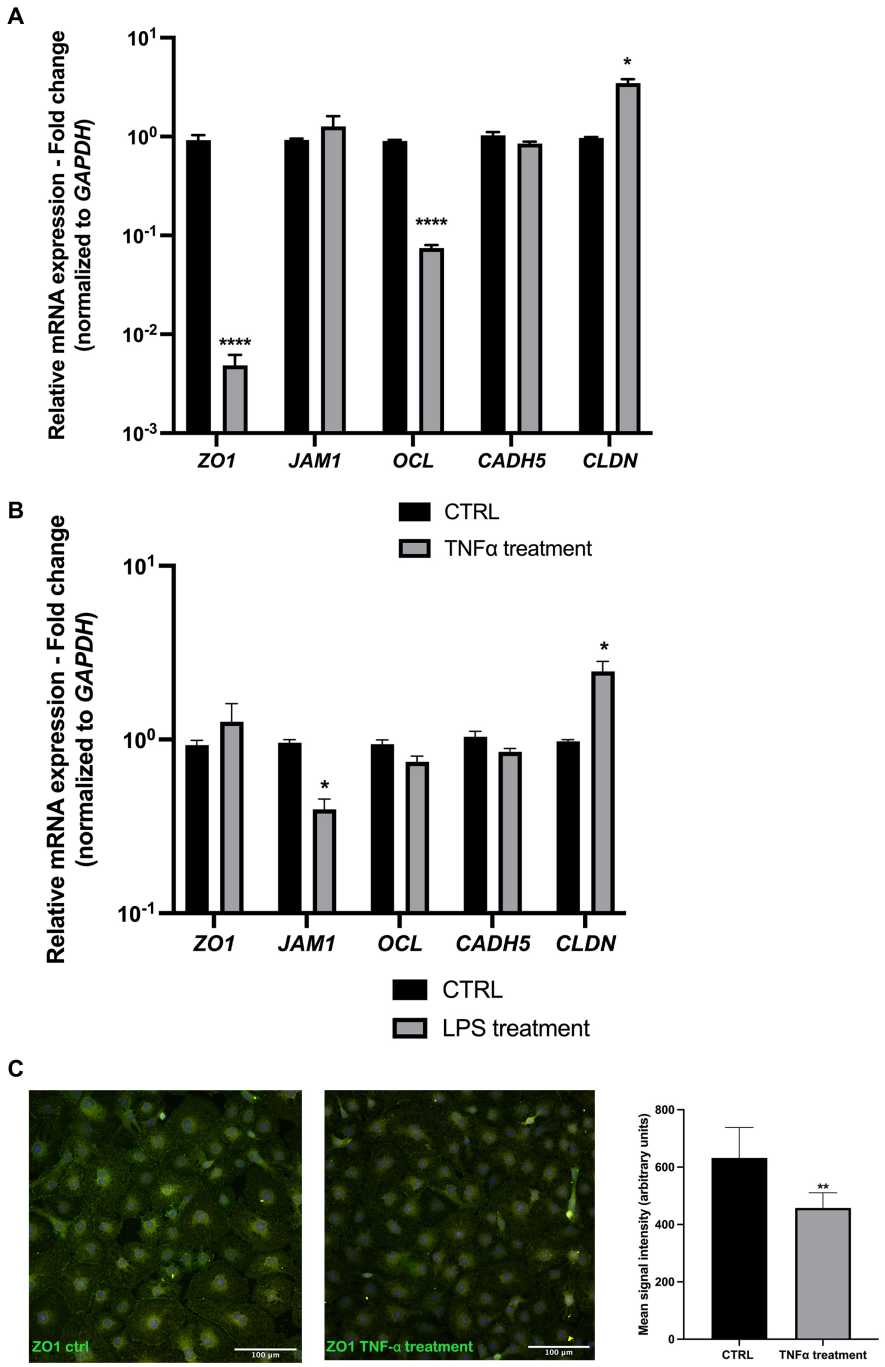
Junctional gene expression is altered upon TNF-α and LPS treatment. **(A)** TNF-α treatment resulted in a significant decrease in *ZO1*, *OCL*, and *CLDN* expression, whereas *JAM1* and *CADH5* levels remained unchanged compared to the control. **(B)** LPS treatment resulted in a slight decrease in *JAM1* and *CLDN* gene expression. **(C)** ZO-1 EC immunostaining shows a decreased signal in TNF-α treated cells. *p < 0.05, **p < 0.01 ****p < 0.0001, Data were obtained from 6 patient-derived cell cultures and done triplicate (technical). *n* = 3. Data are presented as mean ± SD.

As junctional proteins are one of the most important factors in maintaining endothelial blood vessel barrier integrity, following our initial integrity measurements, we exposed the endothelial layer to 50 ng/mL TNF⍺ and 100 ng/mL LPS for 72 h and measured the gene expression levels of some of the major junctional genes. The results showed a significant decrease in *ZO1*, *OCL*, and *CLDN* expression levels in samples exposed to TNF-α (Figure 3A). In LPS-treated samples, a slight decrease in *JAM1* and slight increase in *CLDN* expression was observed (Figure 3B). Upon ZO-1 immunostaining, EC cells exposed to TNF⍺ revealed a lower signal compared to the control (Figure 3C).

### Endothelial barrier permeability is disrupted in the presence of TNF-α

**Figure 4:**
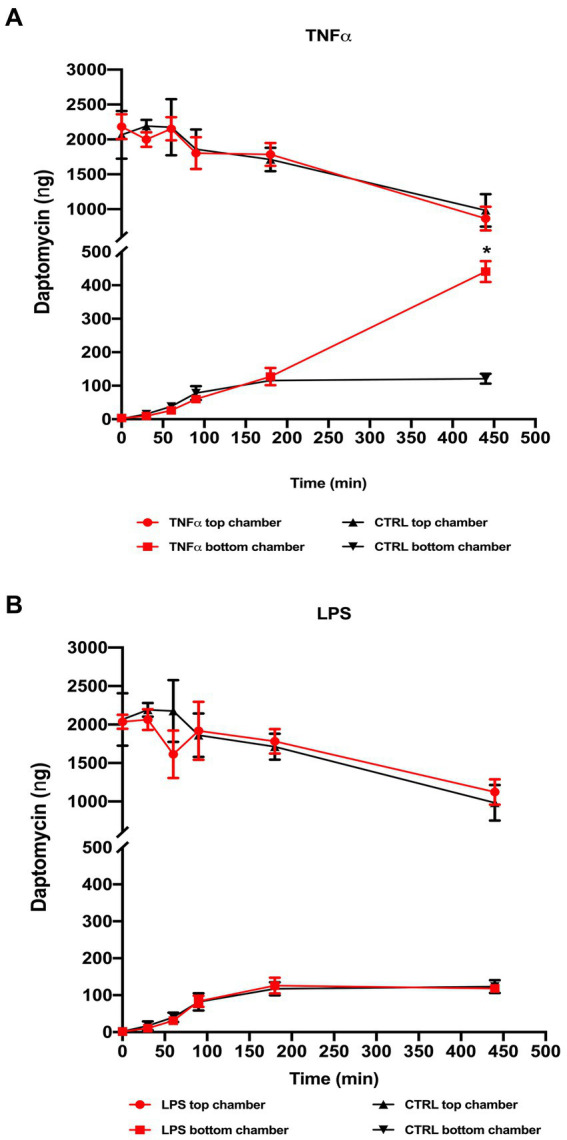
Permeability after daptomycin treatment based on HPLC analysis of the samples collected from the top and bottom chambers. 2 μg daptomycin was added to the top chamber and an aliquot (20 μL) taken from the top and bottom chambers of both the treated and control Transwell at defined time points (0, 30, 60, 90, 180, and 440 min) and compared to control samples. **(A)** TNF-α treatment showed a significant increase in the daptomycin influx at the last measured time point compared to the untreated Transwell, whereas **(B)** LPS treatment resulted in no significant changes in measured daptomycin concentrations between the control and treatment Transwells. *p < 0.05, *n* = 6. Data are presented as mean ± SD.

With previous results indicating barrier changes after exposure to TNF-α and, to a slight extent, LPS, we performed permeability assays using daptomycin (molecular weight 1.6 kDa), which normally does not pass the healthy blood vessel barrier. We added 2000 ng of daptomycin into the top chamber with ECs pre-treated with TNF-α or LPS and measured the concentrations of daptomycin in both chambers at 0, 30, 60, 90, 180, and 440 min and compared them to the control samples. At the initial time points, there was no difference between the control and TNF-α-treated Transwells, and the first significant difference was with daptomycin passing from the top to the bottom chamber at 440 min (Figure 4A). In the LPS pre-treated samples, there was no difference in daptomycin permeability over the measured time (Figure 4B).

### Syndecan1 decrease in the presence of TNF-α

**Figure 5:**
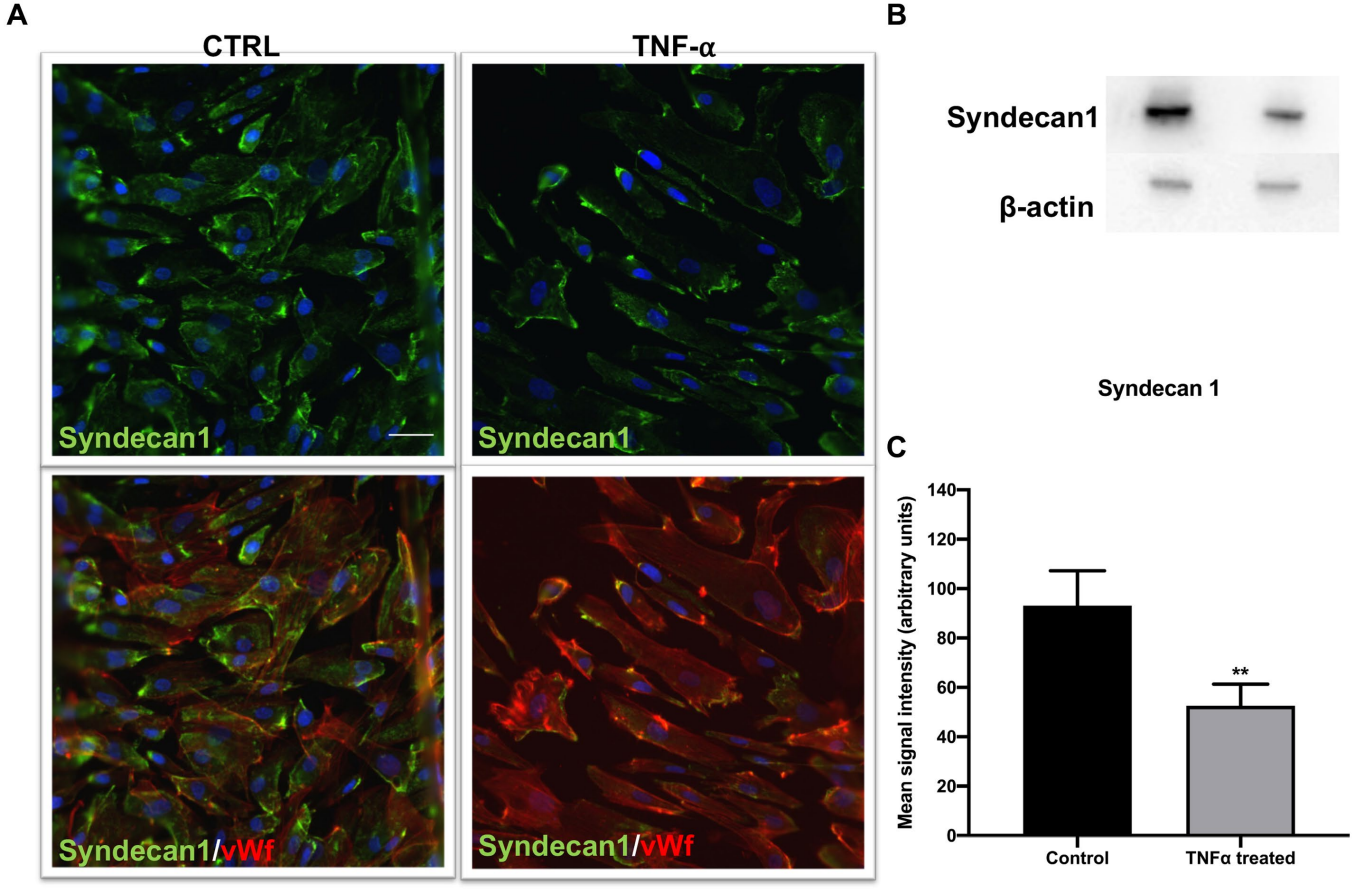
Endothelial marker syndecan1 decreased after TNF-α treatment. **(A)** Cells were immunostained with vWf (red), proteoglycan marker of EC damage; syndecan1 (green); and DAPI (blue). In the TNF-α-treated cells, the decrease in syndecan1 compared to control was observed (top), as well as in the double staining with EC marker vWf (bottom). Scale bar: 100 μm. **(B)** The Western blot signal was visibly weaker in TNF-α-treated cells, which was confirmed by **(C)** signal quantification. **p < 0.01, *n* = 3. Data are presented as mean ± SD.

Considering previous results and a major impact of TNF-α on endothelial integrity, we explored its effect on syndecan1 expression in our culture conditions. Syndecan1 is expressed in ECs and is one of the main markers of endothelial glycocalyx degradation. After exposure to TNF-α, immunostaining revealed a significant decrease in syndecan1 compared with control samples (Figures 5A,B). The immunostaining results were confirmed by decreased syndecan1 protein levels in TNF-α-treated samples.

## Discussion

Loss of BLB integrity is found in acoustic trauma, autoimmune inner ear disease, MD, and presbycusis. MD is a serious condition characterized by fluctuating hearing loss, episodic vertigo, and ear fullness, whereas the etiology and pathophysiology are currently poorly understood (Hallpike and Cairns, 1938). Examinations in patients with MD using MRI have revealed that the degree of hydrops positively corelates with the degree of hearing loss (Sepahdari et al., 2015).

On the other hand, elevated levels of pro-inflammatory cytokines have been found in a subset of patients suffering from MD (Frejo et al., 2018). Changes in BLB physiology have been mentioned as a culprit in MD, but only a few studies have been performed in humans. A major reason is inaccessibility of the human cochlear tissue. Though BLB has been studied in detail in animal models, no adequate BLB human studies have been performed. In addition, MD is primarily a human disease and, therefore, no animal models mimic this condition. This represents a major drawback in uncovering the causes and possible therapeutic routes for MD patients.

To address this gap, we established the initial Transwell model using human BLB-derived primary ECs and PCs in order to recreate the BLB *in vitro* (Figure 1B). We previously showed that the mouse BLB model can be used as a tool for permeability studies (Sekulic-Jablanovic et al., 2022). As BLB plays a crucial role in maintaining the fine-tuned fluid balance in the inner ear while being a highly selective barrier to many substances, we initially confirmed the functionality of the barrier by using a compound called daptomycin that normally would not pass though the intact barrier due to its 1,619 Da size, as the BLB permeability is in the range of less than 400–600 Da (Supplementary Figure S1). Compared to other studies in similar models that use molecules in the size range 3–40 kDa to assess the integrity of the created barrier and tight junction permeability, here we have employed a molecule that is closer to the real permeability size limit (Harding et al., 2022; Pérez-López et al., 2023). Based on the previously published findings of increased basal levels of some of the pro-inflammatory cytokines (i.e., TNF-α, IL-1β, and IL-6) in a subset of MD patients, we proceeded to test the cytokines or their triggering compound effects on BLB integrity in our Transwell model. We found that TNF-α exhibited the highest negative influence on barrier integrity measured by TEER, followed by IL-6 and LPS, respectively. These were promising results, as upregulation in the expression of TNF-α and IL-6 was found in the endolymphatic sac of patients with unilateral MD (Huang et al., 2022).

Furthermore, considering that the major contributor to barrier integrity is junctional proteins forming closely associated tight and adherens junctions, which mediate and control cell contact integrity and molecular permeability across the endothelial barrier, we investigated the gene expression of some of the genes belonging to this group. A decrease in *ZO1*, *OCL*, and *CLDN5* expression was detected after ECs were exposed to TNF-α, and *JAM* and *CLDN5* were affected to a lower extent upon LPS treatment. In addition, decreased ZO-1 immunostaining signal in TNF-α exposed ECs was detected. These results indicate higher susceptibility to damage of tight junctions under the influence of TNF-α and are in line with our barrier integrity measurements.

We also investigated the levels of one of the biomarkers of endothelial function and glycocalyx degradation, syndecan1, after TNF-α exposure. We found a decrease in both the immunofluorescence signal for syndecan1 and protein content on Western blot in samples treated with TNF-α. As our measurements showed a loss of integrity and decrease in junctional gene expression after TNF-α exposure, we investigated its effect on permeability using daptomycin. We found a significant influx of daptomycin after the cells were exposed to TNF-α, whereas no changes in permeability compared to control were noted in the LPS-treated Transwells. Though LPS has been found to increase BLB permeability in mice, the lack of a similar effect in our system could be due to several factors, such as exposure time, concentration, or even lack of significant impact in human cells (Koo et al., 2015).

These results are promising as a comparative study of MD and ISSNHL found that MD patients have increased BLB permeability, which is a feature that is clearly associated with MD (Pakdaman et al., 2016). More interestingly, the same study found, in the subset of patients with MD without MRI evidence of hydrops, markedly increased BLB permeability was present, which would indicate that hydrops is not necessarily causing defects in the stria vascularis and subsequently affecting the permeability of blood vessels in all MD cases. Therefore, other factors may be influencing BLB permeability. The role of genetic component in inflammatory response has been documented through discovery of allelic variations in the *TLR10* gene that may influence bilateral affectation and clinical course in MD and variants in the *NFKB1* gene that influence the hearing outcome in patients with unilateral MD through suspected mediation of the interaction of *NFKB1* with other transcription factors conditioning the inflammatory response in the inner ear (Requena et al., 2013; Cabrera et al., 2014). Increased levels of IgE, IL-4, IL-5, IL-10, and IL-13 were found in serum samples from MD patients, with close to third of those patients having high basal levels of IgE when compared to controls. The same study indicates a possible role of IgE in initiating inflammation in inner ear through CD23-mediated transcytosis (Zhang et al., 2022). These findings are expanding the array of possible culprits of inflammation in the inner ear. Thus, the need for developing human models to elucidate further the mechanisms behind hearing loss is growing.

## Limitations and future development

Here, we showed several aspects that could lead to damage to the BLB. The major challenge is the long-term maintenance of the human-derived primary cells to recreate the gradual effects of the above-tested compounds over time. One of the ways we aim to overcome this challenge and maintain cells in optimal conditions over extended periods is to seed cells in a dynamic environment, such as the microfluidic device we are currently developing that will mimic blood flow and recreate an *in vivo* environment.

## Conclusion

To date, no human-derived BLB primary cells have been used to create a BLB model, and this is the first step towards establishing a functional human BLB model that mimics possible events occurring in the strial microvasculature upon exposure to inflammatory factors. Having a functional human BLB model is a great step towards understanding normal BLB physiology in humans, as well as enabling the development and testing of new drug therapies for hearing damage in diseases and conditions where the BLB is affected.

## Data availability statement

The raw data supporting the conclusions of this article will be made available by the authors, without undue reservation.

## Ethics statement

The studies involving humans were approved by Ethical permit: EKNZ 2020-01379. The studies were conducted in accordance with the local legislation and institutional requirements. The participants/donors provided their written informed consent to participate in this study.

## Author contributions

MS performed experiments, analyzed data, and drafted the article. RP performed experiments, helped with design, data analysis, and methods writing. VP conceptualized the experimental flow and contributed the article writing. DB provided clinical aspects to the experimental design and manuscript writing. All authors contributed to the article and approved the submitted version.

## Funding

The authors declare that this study received funding from Freiwillige Akademische Gesellschaft. The funder was not involved in the study design, collection, analysis, interpretation of data, the writing of this article, or the decision to submit it for publication.

## Conflict of interest

The authors declare that the research was conducted in the absence of any commercial or financial relationships that could be construed as a potential conflict of interest.

## Publisher’s note

All claims expressed in this article are solely those of the authors and do not necessarily represent those of their affiliated organizations, or those of the publisher, the editors and the reviewers. Any product that may be evaluated in this article, or claim that may be made by its manufacturer, is not guaranteed or endorsed by the publisher.
