# Supplementary figures and images for: Human blood-labyrinth barrier model to study the effects of cytokines and inflammation
Source: Front Mol Neurosci. 2023 Sep 21;16:1243370. doi: 10.3389/fnmol.2023.1243370 (PMC10551159; doi:10.3389/fnmol.2023.1243370)

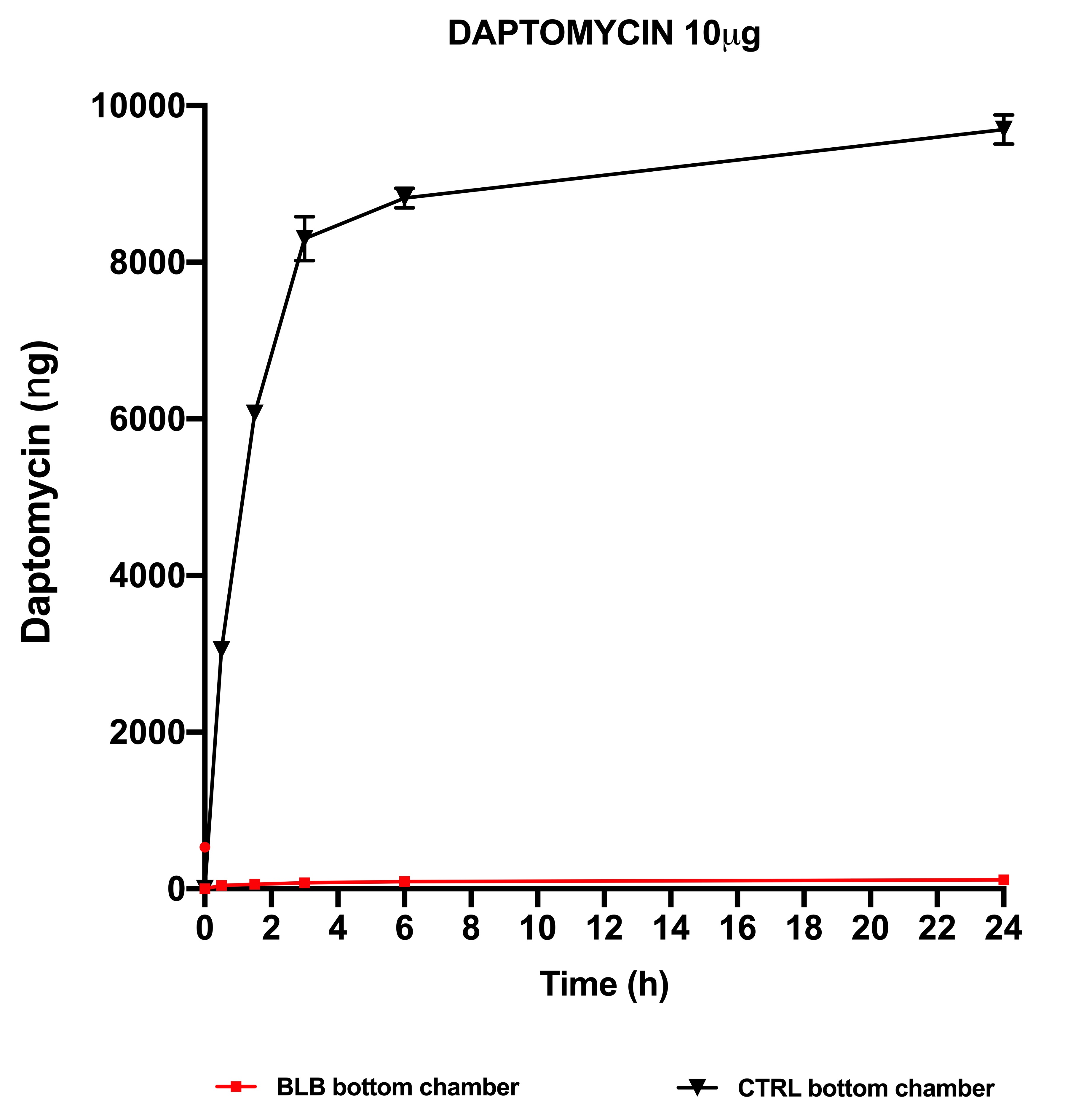

Supplement: Supplementary file 1 [file Image_1.JPEG]
